# Crystal structure of *catena*-poly[[[tetra­aqua­zinc(II)]-μ-1,4-bis­[4-(1*H*-imidazol-1-yl)benzo­yl]piperazine] dinitrate monohydrate]

**DOI:** 10.1107/S2056989015007719

**Published:** 2015-04-25

**Authors:** Chen Hou, Hong-Mei Gan, Jia-Cheng Liu

**Affiliations:** a College of Chemistry and Chemical Engineering, Northwest Normal University, Lanzhou 730070, People’s Republic of China

**Keywords:** crystal structure, zinc complex, one-dimensional coordination polymer, piperazine

## Abstract

In the title polymeric complex, {[Zn(C_24_H_22_N_6_O_2_)(H_2_O)_4_](NO_3_)_2_·2H_2_O}_*n*_, the Zn^II^ cation, located about a twofold rotation axis, is coordinated by two imidazole groups and four water mol­ecules in a distorted N_2_O_4_ octa­hedral geometry; among the four coordinate water mol­ecules, two are located on the same twofold rotation axis. The 1,4-bis­[4-(1*H*-imidazol-1-yl)benzo­yl]piperazine] ligand is centro-symmetric, with the centroid of the piperazine ring located on an inversion center, and bridges the Zn^II^ cations, forming polymeric chains propagating along [201]. In the crystal, O—H⋯O and weak C—H⋯O hydrogen bonds link the polymeric chains, nitrate anions and solvent water mol­ecules into a three-dimensional supra­molecular architecture. A short O⋯O contact of 2.823 (13) Å is observed between neighboring nitrate anions.

## Related literature   

For related coordination polymers and their potential applications, see: Xu *et al.* (2004 ▸); Gandolfo & LaDuca (2011*a* ▸,*b* ▸); Wang *et al.* (2011 ▸, 2014 ▸).
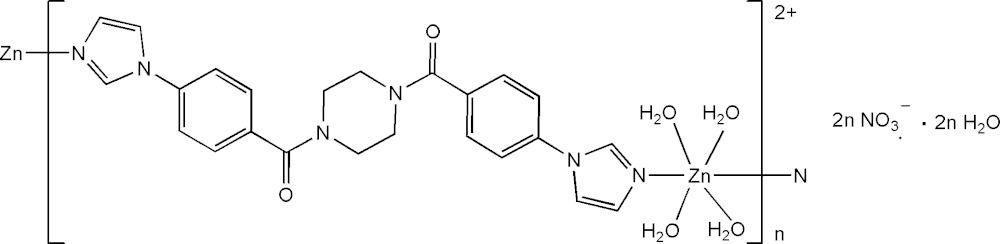


## Experimental   

### Crystal data  

[Zn(C_24_H_22_N_6_O_2_)(H_2_O)_4_](NO_3_)_2_·2H_2_O
*M* *_r_* = 723.96
Monoclinic, 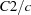

*a* = 22.051 (4) Å
*b* = 7.8861 (16) Å
*c* = 17.837 (4) Å
β = 102.65 (3)°
*V* = 3026.5 (11) Å^3^
*Z* = 4
Mo *K*α radiation
μ = 0.90 mm^−1^
*T* = 294 K
0.27 × 0.25 × 0.22 mm

### Data collection  

Bruker APEXII CCD diffractometer
Absorption correction: multi-scan (*SADABS*; Bruker, 2008 ▸) *T*_min_ = 0.79, *T*_max_ = 0.83
5133 measured reflections
2720 independent reflections
2212 reflections with *I* > 2σ(*I*)
*R*_int_ = 0.030

### Refinement  

*R*[*F* ^2^ > 2σ(*F* ^2^)] = 0.055
*wR*(*F* ^2^) = 0.144
*S* = 1.05
2720 reflections
196 parameters
6 restraints
H-atom parameters constrained
Δρ_max_ = 0.57 e Å^−3^
Δρ_min_ = −0.83 e Å^−3^

### 

Data collection: *APEX2* (Bruker, 2008 ▸); cell refinement: *SAINT* (Bruker, 2008 ▸); data reduction: *SAINT*; program(s) used to solve structure: *SHELXTL* (Sheldrick, 2008 ▸); program(s) used to refine structure: *SHELXTL*; molecular graphics: *SHELXTL*; software used to prepare material for publication: *SHELXTL*.

## Supplementary Material

Crystal structure: contains datablock(s) I, New_Global_Publ_Block. DOI: 10.1107/S2056989015007719/xu5847sup1.cif

Structure factors: contains datablock(s) I. DOI: 10.1107/S2056989015007719/xu5847Isup2.hkl

**Fig. 1.**
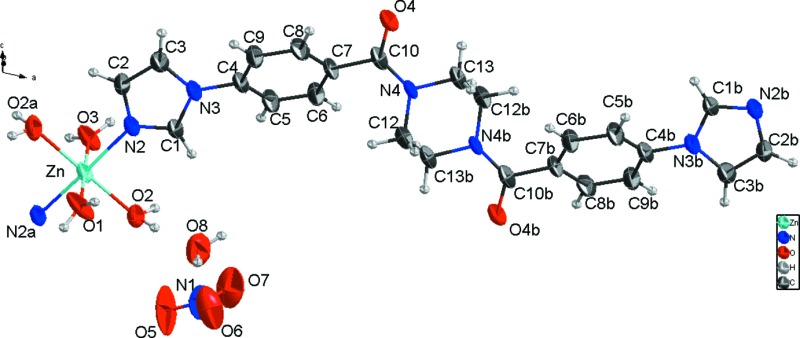
A part of the polymeric chain of the title compound. Displacement ellipsoids are drawn at the 30% probability level.

. DOI: 10.1107/S2056989015007719/xu5847fig1.tif
A part of the polymeric chain of the title compound. Displacement ellipsoids are drawn at the 30% probability level.

CCDC reference: 1060401

Additional supporting information:  crystallographic information; 3D view; checkCIF report

## Figures and Tables

**Table 1:** Hydrogen-bond geometry (, )

*D*H*A*	*D*H	H*A*	*D* *A*	*D*H*A*
O1H1*A*O4^i^	0.84	1.96	2.795(6)	174
O2H2*A*O5^ii^	0.84	2.16	2.956(15)	159
O2H2*A*O6^ii^	0.84	2.32	3.060(10)	148
O2H2*B*O8	0.85	1.83	2.676(7)	176
O3H3*A*O4^iii^	0.84	1.99	2.804(6)	163
O8H8*A*O7	0.85	2.14	2.914(11)	151
O8H8*B*O7^iv^	0.82	2.14	2.926(11)	160
C1H1O6^ii^	0.93	2.51	3.232(11)	135
C5H5O6^ii^	0.93	2.59	3.485(12)	162

Symmetry codes: (i) 
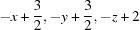
; (ii) 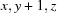; (iii) 
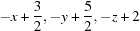
; (iv) 
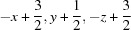
.

## supplementary crystallographic information

### S1. Structural commentary

Piperazine derivatives as N-containing ligands which have different combination of span, multi-coordination point and hydrogen-bonding points of contact, to conform coordination complexes has attracted great attention. (Wang *et al.*, 2011, 2014; Gandolfo & LaDuca *et al.*, 2011a,b; Xu *et al.*, 2004). Nevertheless, piperazine derivatives-containing the imidazole group as the coordinated point have been designed forming coordination compounds relatively few. As the imidazole has a similar properties with pyridine, so in this context, we design and successfully synthesized the compound {[Zn(C_24_H_22_N_6_O_2_)(H_2_O)_4_](NO_3_)_2_(H_2_O)_2_}_n_ based on the piperazine-1,4-diylbis((4-(1H-imidazol-1-yl)phenyl)­methanone) ligand, obtained under hydro­thermal technique. An asymmetric unit of the title compound includes a half Zn^II^, a half piperazine-1,4-diylbis((4-(1H-imidazol-1-yl)phenyl)­methanone) ligands, two coordinated water molecules, an uncoordinated nitrate anion and one uncoordinated water molecule (Fig. 1). The Zn^II^ atom is coordinated and lies on an inversion centre of a slighter distorted o­cta­hedral, with two ligands [two imidazole N atoms, Zn—N = 2.122 (3) Å] and four coordinated water molecules [Zn—O bond lengths in the range of 2.112 (4) – 2.138 (3) Å]. Between the 1D chains formed by the ligand and Zn^II^ atoms are inter­connected via water O—H···O hydrogen bonds to form a three dimension supra­molecular architecture. In the crystal, in the nitrate anions and the uncoordinated water molecules forming the O—H···N hydrogen bonds to stabilize the three dimension skeleton (Fig. 2).

### S2. Synthesis and crystallization

A mixture of L (0.1 mmol, 0.0462 g), Zn(NO_3_)_2_.6H_2_O (0.2 mmol, 0.059 g), distilled water (6.0 mL) sealed in a 25 mL Teflon-lined stainless steel vessel and heated at 130 °C for 72 h under auto-pressure, after cooling to room temperature. Primrose yellow prismatic single crystals were removed (yield: 24%).

### S3. Refinement details

Water H atoms were located in a difference Fourier map and refined in riding mode with U_iso_(H) = 1.2U_eq_(O). Other H atoms were placed in calculated positions with C—H = 0.93–0.96 Å and refined using a riding model, *U*_iso_(H) = 1.2*U*_eq_(C).

### Crystal data

**Table:**

[Zn(C_24_H_22_N_6_O_2_)(H_2_O)_4_](NO_3_)_2_·2H_2_O	*F*(000) = 1504
*M**_r_* = 723.96	*D*_x_ = 1.589 Mg m^−^^3^
Monoclinic, *C*2/*c*	Mo *K*α radiation, λ = 0.71073 Å
Hall symbol: -C 2yc	Cell parameters from 7728 reflections
*a* = 22.051 (4) Å	θ = 2.7–28.6°
*b* = 7.8861 (16) Å	µ = 0.90 mm^−^^1^
*c* = 17.837 (4) Å	*T* = 294 K
β = 102.65 (3)°	Block, colorless
*V* = 3026.5 (11) Å^3^	0.27 × 0.25 × 0.22 mm
*Z* = 4	

### Data collection

**Table:**

Bruker APEXII CCD diffractometer	2720 independent reflections
Radiation source: fine-focus sealed tube	2212 reflections with *I* > 2σ(*I*)
Graphite monochromator	*R*_int_ = 0.030
ω scan	θ_max_ = 25.2°, θ_min_ = 3.3°
Absorption correction: multi-scan (*SADABS*; Bruker, 2008)	*h* = −26→26
*T*_min_ = 0.79, *T*_max_ = 0.83	*k* = −9→6
5133 measured reflections	*l* = −21→11

### Refinement

**Table:**

Refinement on *F*^2^	Primary atom site location: structure-invariant direct methods
Least-squares matrix: full	Secondary atom site location: difference Fourier map
*R*[*F*^2^ > 2σ(*F*^2^)] = 0.055	Hydrogen site location: inferred from neighbouring sites
*wR*(*F*^2^) = 0.144	H-atom parameters constrained
*S* = 1.05	*w* = 1/[σ^2^(*F*_o_^2^) + (0.0575*P*)^2^ + 8.9494*P*] where *P* = (*F*_o_^2^ + 2*F*_c_^2^)/3
2720 reflections	(Δ/σ)_max_ < 0.001
196 parameters	Δρ_max_ = 0.57 e Å^−^^3^
6 restraints	Δρ_min_ = −0.83 e Å^−^^3^

### Special details

**Table:**

Geometry. All esds (except the esd in the dihedral angle between two l.s. planes) are estimated using the full covariance matrix. The cell esds are taken into account individually in the estimation of esds in distances, angles and torsion angles; correlations between esds in cell parameters are only used when they are defined by crystal symmetry. An approximate (isotropic) treatment of cell esds is used for estimating esds involving l.s. planes.
Refinement. Refinement of F^2^ against ALL reflections. The weighted R-factor wR and goodness of fit S are based on F^2^, conventional R-factors R are based on F, with F set to zero for negative F^2^. The threshold expression of F^2^ > 2sigma(F^2^) is used only for calculating R-factors(gt) etc. and is not relevant to the choice of reflections for refinement. R-factors based on F^2^ are statistically about twice as large as those based on F, and R- factors based on ALL data will be even larger.

### Fractional atomic coordinates and isotropic or equivalent isotropic displacement parameters (Å^2^)

**Table:**

	*x*	*y*	*z*	*U*_iso_*/*U*_eq_	
Zn1	0.5000	0.96013 (12)	0.7500	0.0363 (3)	
N1	0.6774 (3)	0.2834 (11)	0.7201 (4)	0.078 (2)	
N2	0.56556 (19)	0.9716 (6)	0.8564 (2)	0.0366 (10)	
N3	0.6522 (2)	0.9960 (6)	0.9451 (3)	0.0380 (11)	
O1	0.5000	0.6945 (7)	0.7500	0.0656 (19)	
H1A	0.5215	0.6270	0.7813	0.079*	
O2	0.57119 (17)	0.9622 (6)	0.6871 (2)	0.0541 (11)	
H2A	0.5960	1.0433	0.6903	0.065*	
H2B	0.5993	0.8872	0.6922	0.065*	
O3	0.5000	1.2290 (7)	0.7500	0.0530 (15)	
H3A	0.5283	1.2929	0.7738	0.064*	
O4	0.92408 (18)	1.0374 (6)	1.1542 (2)	0.0508 (11)	
O5	0.6282 (4)	0.3010 (19)	0.6852 (6)	0.224 (7)	
O6	0.6930 (4)	0.1535 (12)	0.7462 (6)	0.164 (4)	
O7	0.7160 (4)	0.3959 (12)	0.7332 (7)	0.167 (4)	
O8	0.6636 (2)	0.7354 (8)	0.7064 (4)	0.104 (2)	
H8A	0.6651	0.6280	0.7091	0.125*	
H8B	0.6952	0.7741	0.7339	0.125*	
C1	0.6268 (2)	0.9722 (8)	0.8698 (3)	0.0410 (13)	
H1	0.6496	0.9579	0.8321	0.049*	
C2	0.5516 (3)	0.9983 (8)	0.9263 (3)	0.0466 (15)	
H2	0.5115	1.0051	0.9347	0.056*	
C3	0.6038 (3)	1.0134 (9)	0.9812 (3)	0.0499 (16)	
H3	0.6065	1.0320	1.0333	0.060*	
C4	0.7176 (2)	1.0017 (7)	0.9795 (3)	0.0380 (13)	
C5	0.7578 (2)	1.0726 (8)	0.9392 (3)	0.0426 (14)	
H5	0.7429	1.1197	0.8909	0.051*	
C6	0.8208 (2)	1.0726 (8)	0.9719 (3)	0.0440 (14)	
H6	0.8484	1.1186	0.9447	0.053*	
C7	0.8435 (2)	1.0051 (7)	1.0444 (3)	0.0381 (13)	
C8	0.8017 (3)	0.9408 (8)	1.0846 (3)	0.0445 (14)	
H8	0.8161	0.9001	1.1343	0.053*	
C9	0.7385 (3)	0.9360 (8)	1.0522 (3)	0.0447 (14)	
H9	0.7108	0.8893	1.0789	0.054*	
C10	0.9109 (3)	1.0080 (7)	1.0844 (3)	0.042	
N4	0.95517 (18)	0.9803 (6)	1.0449 (2)	0.036	
C12	0.9476 (3)	0.8984 (9)	0.9696 (3)	0.050	
H12A	0.9037	0.8881	0.9463	0.060*	
H12B	0.9651	0.7851	0.9762	0.060*	
C13	1.0212 (3)	1.0026 (9)	1.0821 (4)	0.0522 (16)	
H13A	1.0410	0.8927	1.0926	0.063*	
H13B	1.0248	1.0620	1.1305	0.063*	

### Atomic displacement parameters (Å^2^)

**Table:**

	*U*^11^	*U*^22^	*U*^33^	*U*^12^	*U*^13^	*U*^23^
Zn1	0.0232 (5)	0.0463 (6)	0.0345 (5)	0.000	−0.0042 (3)	0.000
N1	0.057 (4)	0.099 (6)	0.076 (4)	0.007 (4)	0.010 (4)	0.036 (4)
N2	0.024 (2)	0.047 (3)	0.034 (2)	0.0000 (19)	−0.0029 (18)	−0.001 (2)
N3	0.023 (2)	0.054 (3)	0.033 (2)	−0.0007 (19)	−0.0043 (18)	−0.001 (2)
O1	0.069 (4)	0.042 (3)	0.063 (4)	0.000	−0.035 (3)	0.000
O2	0.027 (2)	0.086 (3)	0.047 (2)	0.004 (2)	0.0023 (18)	−0.005 (2)
O3	0.047 (3)	0.044 (3)	0.054 (4)	0.000	−0.019 (3)	0.000
O4	0.033 (2)	0.076 (3)	0.037 (2)	−0.001 (2)	−0.0075 (17)	−0.008 (2)
O5	0.069 (5)	0.422 (19)	0.157 (8)	0.008 (7)	−0.027 (5)	0.171 (10)
O6	0.126 (7)	0.130 (7)	0.210 (10)	−0.035 (6)	−0.019 (6)	0.073 (7)
O7	0.127 (7)	0.104 (6)	0.276 (13)	−0.019 (6)	0.054 (8)	0.027 (7)
O8	0.055 (3)	0.113 (5)	0.137 (6)	0.025 (3)	0.004 (3)	−0.029 (4)
C1	0.025 (3)	0.063 (4)	0.031 (3)	0.003 (3)	−0.002 (2)	−0.006 (3)
C2	0.024 (3)	0.073 (4)	0.041 (3)	0.000 (3)	0.003 (2)	−0.004 (3)
C3	0.034 (3)	0.080 (5)	0.034 (3)	−0.005 (3)	0.004 (2)	−0.004 (3)
C4	0.025 (3)	0.050 (3)	0.033 (3)	−0.001 (2)	−0.006 (2)	−0.001 (2)
C5	0.031 (3)	0.057 (4)	0.033 (3)	−0.002 (3)	−0.005 (2)	0.009 (3)
C6	0.029 (3)	0.061 (4)	0.040 (3)	−0.006 (3)	0.002 (2)	0.007 (3)
C7	0.024 (3)	0.050 (3)	0.035 (3)	−0.001 (2)	−0.007 (2)	−0.003 (2)
C8	0.037 (3)	0.059 (4)	0.031 (3)	0.000 (3)	−0.007 (2)	0.005 (3)
C9	0.029 (3)	0.065 (4)	0.037 (3)	−0.005 (3)	0.002 (2)	0.006 (3)
C10	0.029	0.049	0.040	−0.001	−0.010	0.002
N4	0.018	0.055	0.029	−0.006	−0.006	−0.009
C12	0.030	0.063	0.050	−0.006	−0.004	−0.014
C13	0.027 (3)	0.079 (5)	0.043 (3)	−0.005 (3)	−0.009 (2)	−0.011 (3)

### Geometric parameters (Å, º)

**Table:**

Zn1—O1	2.095 (6)	C2—H2	0.9300
Zn1—O2^i^	2.120 (4)	C3—H3	0.9300
Zn1—O2	2.120 (4)	C4—C5	1.375 (8)
Zn1—O3	2.120 (6)	C4—C9	1.379 (8)
Zn1—N2	2.121 (4)	C5—C6	1.384 (7)
Zn1—N2^i^	2.121 (4)	C5—H5	0.9300
N1—O5	1.136 (9)	C6—C7	1.387 (8)
N1—O6	1.146 (10)	C6—H6	0.9300
N1—O7	1.216 (10)	C7—C8	1.383 (8)
N2—C1	1.318 (7)	C7—C10	1.501 (7)
N2—C2	1.364 (7)	C8—C9	1.386 (8)
N3—C1	1.351 (7)	C8—H8	0.9300
N3—C3	1.369 (7)	C9—H9	0.9300
N3—C4	1.439 (6)	C10—N4	1.342 (7)
O1—H1A	0.8391	N4—C12	1.466 (7)
O2—H2A	0.8351	N4—C13	1.471 (6)
O2—H2B	0.8465	C12—C13^ii^	1.489 (9)
O3—H3A	0.8409	C12—H12A	0.9700
O4—C10	1.235 (7)	C12—H12B	0.9700
O8—H8A	0.8490	C13—C12^ii^	1.489 (9)
O8—H8B	0.8196	C13—H13A	0.9700
C1—H1	0.9300	C13—H13B	0.9700
C2—C3	1.344 (8)		
			
O1—Zn1—O2^i^	90.45 (13)	N3—C3—H3	126.8
O1—Zn1—O2	90.45 (13)	C5—C4—C9	121.5 (5)
O2^i^—Zn1—O2	179.1 (3)	C5—C4—N3	119.4 (5)
O1—Zn1—O3	180.000 (3)	C9—C4—N3	119.1 (5)
O2^i^—Zn1—O3	89.55 (13)	C4—C5—C6	118.8 (5)
O2—Zn1—O3	89.55 (13)	C4—C5—H5	120.6
O1—Zn1—N2	92.45 (13)	C6—C5—H5	120.6
O2^i^—Zn1—N2	87.99 (16)	C5—C6—C7	121.1 (5)
O2—Zn1—N2	91.97 (16)	C5—C6—H6	119.4
O3—Zn1—N2	87.55 (13)	C7—C6—H6	119.4
O1—Zn1—N2^i^	92.45 (13)	C8—C7—C6	118.6 (5)
O2^i^—Zn1—N2^i^	91.97 (16)	C8—C7—C10	117.5 (5)
O2—Zn1—N2^i^	87.99 (16)	C6—C7—C10	123.8 (5)
O3—Zn1—N2^i^	87.55 (13)	C7—C8—C9	121.1 (5)
N2—Zn1—N2^i^	175.1 (3)	C7—C8—H8	119.5
O5—N1—O6	119.8 (11)	C9—C8—H8	119.5
O5—N1—O7	124.1 (11)	C4—C9—C8	118.7 (5)
O6—N1—O7	116.1 (9)	C4—C9—H9	120.6
C1—N2—C2	105.2 (4)	C8—C9—H9	120.6
C1—N2—Zn1	129.2 (4)	O4—C10—N4	121.4 (5)
C2—N2—Zn1	125.3 (4)	O4—C10—C7	118.2 (5)
C1—N3—C3	106.5 (4)	N4—C10—C7	120.4 (5)
C1—N3—C4	125.9 (5)	C10—N4—C12	127.1 (4)
C3—N3—C4	127.5 (5)	C10—N4—C13	120.4 (4)
Zn1—O1—H1A	129.4	C12—N4—C13	111.6 (4)
Zn1—O2—H2A	121.3	N4—C12—C13^ii^	111.2 (5)
Zn1—O2—H2B	123.2	N4—C12—H12A	109.4
H2A—O2—H2B	94.3	C13^ii^—C12—H12A	109.4
Zn1—O3—H3A	126.8	N4—C12—H12B	109.4
H8A—O8—H8B	108.6	C13^ii^—C12—H12B	109.4
N2—C1—N3	111.5 (5)	H12A—C12—H12B	108.0
N2—C1—H1	124.3	N4—C13—C12^ii^	109.3 (5)
N3—C1—H1	124.3	N4—C13—H13A	109.8
C3—C2—N2	110.5 (5)	C12^ii^—C13—H13A	109.8
C3—C2—H2	124.8	N4—C13—H13B	109.8
N2—C2—H2	124.8	C12^ii^—C13—H13B	109.8
C2—C3—N3	106.3 (5)	H13A—C13—H13B	108.3
C2—C3—H3	126.8		

Symmetry codes: (i) −*x*+1, *y*, −*z*+3/2; (ii) −*x*+2, −*y*+2, −*z*+2.

### Hydrogen-bond geometry (Å, º)

**Table:**

*D*—H···*A*	*D*—H	H···*A*	*D*···*A*	*D*—H···*A*
O1—H1*A*···O4^iii^	0.84	1.96	2.795 (6)	174
O2—H2*A*···O5^iv^	0.84	2.16	2.956 (15)	159
O2—H2*A*···O6^iv^	0.84	2.32	3.060 (10)	148
O2—H2*B*···O8	0.85	1.83	2.676 (7)	176
O3—H3*A*···O4^v^	0.84	1.99	2.804 (6)	163
O8—H8*A*···O7	0.85	2.14	2.914 (11)	151
O8—H8*B*···O7^vi^	0.82	2.14	2.926 (11)	160
C1—H1···O6^iv^	0.93	2.51	3.232 (11)	135
C5—H5···O6^iv^	0.93	2.59	3.485 (12)	162

Symmetry codes: (iii) −*x*+3/2, −*y*+3/2, −*z*+2; (iv) *x*, *y*+1, *z*; (v) −*x*+3/2, −*y*+5/2, −*z*+2; (vi) −*x*+3/2, *y*+1/2, −*z*+3/2.
