# Supplementary figures and images for: Crystal structure of catena-poly[[[tetra­aqua­zinc(II)]-μ-1,4-bis­[4-(1H-imidazol-1-yl)benzo­yl]piperazine] dinitrate monohydrate]
Source: Acta Crystallogr E Crystallogr Commun. 2015 Apr 25;71(Pt 5):m120–1. doi: 10.1107/S2056989015007719 (PMC4420132; doi:10.1107/S2056989015007719)

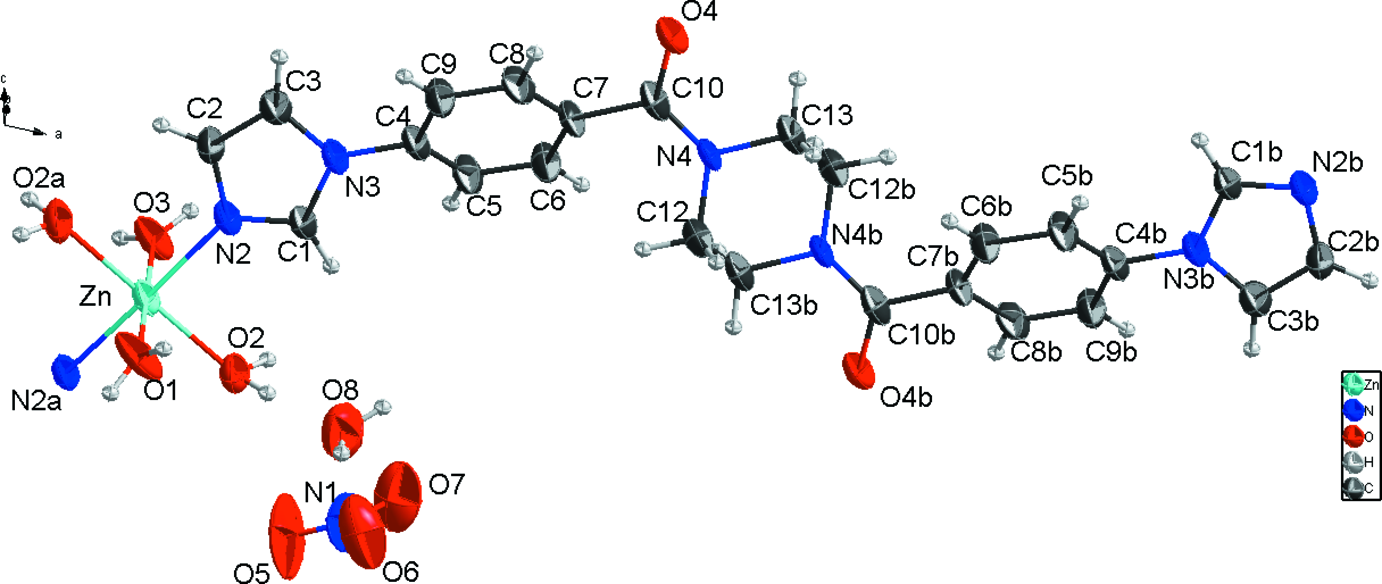

Supplement: Supplementary file 3 [file e-71-0m120-fig1.tif]
